# Assessment of Bax and Bcl-2 Immunoexpression in Patients with Oral Lichen Planus and Oral Squamous Cell Carcinoma

**Published:** 2013

**Authors:** Shima Nafarzadeh, Sina Jafari, Ali Bijani

**Affiliations:** 1 *Dental Material Research Center, Dental Faculty, Babol University of Medical Sciences and Health Services, Babol, Iran.*; 2 *Department of Oral and Maxillofacial Pathology, Dental Faculty, Babol University of Medical Sciences and Health Services, Babol, Iran.*; 3 *Student Research Committee, Dental Faculty, Babol University of Medical Sciences and Health Services, Babol, Iran.*; 4 *Non-Communicable Pediatric Diseases Research Centre, Babol University of Medical Sciences and Health Services, Babol, Iran.*

**Keywords:** Oral Lichen Planus, oral Squamous Cell Carcinoma, apoptosis

## Abstract

Lichen planus (LP) is a chronic inflammatory disease of probable immune-based etiology. The pathogenesis of LP is unclear, but apoptotic changes in epidermal (epithelial) cells have been reported. Destruction of the basal cell layer is observed and many changes in cell proliferation, cell repair and cell death occur in the injured mucosal epithelium. The aim of this study was to evaluate and compare the expression of bax and bcl-2 in oral lichen planus (OLP), well differentiated oral squamous cell carcinoma (WOSCC) and normal mucosa. Sixty one paraffin-embedded biopsy including 11 cases of WOSCC, 30 cases of OLP (n=15 erosive OLP [OLP-E], n=15 reticular OLP [OLP-R]) and 20 normal mucosa were entered in our research. We used immunohistochemistry staining method for assessing bax and bcl-2 expression in epithelial layers. The percentage of stained cells was estimated in 5 randomized microscopic fields and classified as (-): 0%, (+) :< 10%, (++): 10-25%, (+++): 26-50%, (++++): > 50% positive cells. The data were analyzed with Mann-Whitney, Chi Square, and Kruskal-Wallis tests. Significant differences in bax expression were observed among OLP, WOSCC compared to normal mucosa (P=0.008). No significant difference in bax expression between OLP-E and OLP-R compared to WOSCC was seen (P>0.05). Bcl-2 was negative for all OLP and normal mucosa samples, and weak positivity was observed in WOSCC samples. According to the findings of our study, it may be possible to correlate the difference of bax and bcl-2 expression levels among the mentioned lesions to the malignant potential of OLP.

RLichen planus (LP) is a chronic inﬂammatory disease of skin and mucosa affecting approximately 1-2% of the adult population (1). Oral lichen planus (OLP) was first described by Wilson in 1869. Despite many studies, the pathogenesis of OLP is still unknown. Clinical and immunohistochemical studies strongly support an immunologic basis for the disease (2). The potential form alignant transformation of OLP, especially erosive type remains controversial.

In 1999, Van der Meij et al. reviewed OLP cases with malignant transformation and concluded that two thirds of these cases, did not present clear criteria regarding the clinical and histopathologic diagnosis of the disease (3). One year later, Eisenberg and Krutchkoff mentioned a lack of criteria for the diagnosis of OLP (4).

Since 2005, the World Health Organization (WHO) classified OLP as a potentially malignant disorder, but it presents a lower risk of malignant transformation compared to leukoplakia and erythroplakia (5). However, such classification is not fully accepted because it is the target of countless debates. Despite several retrospective and prospective epidemiologic studies performed in the last 20 years in several countries, it was shown that 1.63% of initially diagnosed lesions as OLP evolve into oral squamous cell carcinomas (6). Some authors believe that most malignant transformed cases described in the literature, have not been sufficiently documented to be described as such (3, 7-8). Several studies have suggested that OLP has a malignant potential and the rate of malignant transformation in OLP has been estimated to be 0.4- 6.25% (9-10). However, other studies failed to prove the malignant transformation potential of OLP (3, 11).

Apoptosis is a programmed cell death which is induced by cell damage beyond repair, viral infection or stress conditions such as starvation. Apoptosis can be caused by the cell itself, the surrounding tissue or inflammatory cells (12). The triggers for apoptosis in OLP are still unknown, but T cells attack to basal epithelial cells triggers a series of complex molecular mechanisms designed to arrest the cell cycle for DNA repair, induce cell senescence or apoptosis to eliminate cells with severely damaged DNA (13). However, some authors demonstrated that epithelial cells in OLP frequently respond to this attack with an increase in cell proliferation rate (14-15). Recent techniques such as immunohistochemistry and molecular biology, have been contributed to end this polemic subject. Despite being different methodologies, these techniques have a single objective: to evaluate the expression of proteins related to the regulation of the cellular cycle.

It is currently known that alterations in the mechanisms of cell proliferation and apoptosis are essential for carcinogenesis. Alterations in expression and consequently, function of such proteins may be a strong indicator for malignant transformation of a lesion. Among the several proteins involved in cell proliferation and apoptosis processes, bcl-2 and bax deserve greater attention; not only for their known key role in these processes but also for the participation in the human carcinogenesis process especially in oral cavity (16). Detection of apoptotic abnormalities before the consequences become clinically or histologically detectable will greatly help the early diagnosis.

The increase in expression of bcl-2 is not only essential for oral carcinogenesis, but also influences the progression of the disease. Because it increases the survival rate of neoplastic cells, allowing new genetic mutation to occur and granting their higher resistance to chemotherapy and radiotherapy (6, 17). Considering the importance of bcl-2 and bax in apoptosis, and because few researches have evaluated the role of these apoptotic markers in malignant changes of OLP, the aim of this study was to evaluate and compare the expression of bax and bcl-2 in OLP, WOSCC and normal oral mucosa.

## Material and Methods

**Samples**

A total of sixty paraffin-embedded biopsy samples with diagnosis of OLP and OSCC were collected from the archive of oral and maxillofacial pathology department of Babol dental school. The specimens included 30 of OLP, 20 samples of normal mucosa (Periphery of OLP samples) (18) and 11 of WOSCC. The OLP tissues were further classified as: (a) erosive type (n=15) and (b) reticular type (n=15). In order to confirm the diagnosis, Hematoxylin and Eosin-stained slides were re-evaluated before entering the samples in our study.

**Immunohistochemical staining**

Immunohistochemistry procedure was used to detect the expression of bax and bcl-2 markers. Briefly, 4 µm thick sections from formalin-fixed and paraffin-embedded samples were prepared. The peroxidase-antiperoxidase (PAP) technique was used, performing immunohistochemical analysis by means of the avidin-biotin method. Slides were deparaffinized in xylene, rehydrated and incubated with 0.5% (v/v) H_2_O_2_ in methanol for 20 min to block the endogenous peroxidase activity. Slides were then washed with Tris-buffered saline (TBS) and heated for 15 min at 100°C in 10 mM sodium citrate buffer (pH 6.0) and boiled in a microwave oven (700 W) for antigen epitope retrieval. Non-specific binding was blocked by incubation of slides with 1% BSA for 1 h. Sections were incubated with primary antibodies (anti-Bcl-2 antibody dilution 1:100 and anti-Bax antibody dilution 1:100, Sigma-Aldrich, St. Louis, Missouri, USA) overnight at 4 °C, then biotinylated horse antimouse IgG secondary antibodies were used at 1: 200 dilution for 30 min, then visualized using diaminobenzidine (DAB) as chromogen. Slides were washed with TBS after each step. Finally, they were counter-stained with Mayer’s hematoxylin, dehydrated and mounted with DPX mountant. Negative control samples were processed in parallel to the test samples by replacing the primary antibody with phosphate buffer saline (PBS). We used breast carcinoma and normal tonsil tissue as positive control for bax and bcl-2 respectively.

Microscopic examination was performed by a pathologist under Olympus BX41 (Olympus, Tokyo, Japan) light microscope. Expression index was determined based on the percentage of stained cells in basal and parabasal layers in five high power fields. Cases were assigned to one of the following categories: 0% positive cells (-), <10% positive cells (+), 10-25% positive cells (++), 26-50% positive cells (+++) or >50% positive cells (++++) (19). 

**Statistical analysis**

SPSS Version 18.00 was used for statistical analysis. The data were analyzed using the Mann-Whitney, Chi-Square, and Kruskal-Wallis tests.

## Results

**Fig.1:**
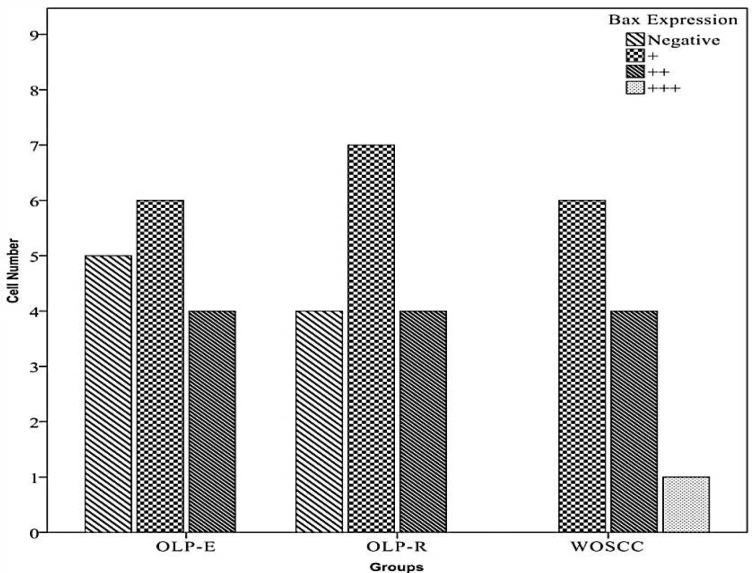
Frequency of Bax positive cells among OLP-E, OLP-R and WOSCC

Significant differences in bax expression were observed among OLP and WOSCC compared to normal mucosa (P=0.008). No significant diff-erence in bax expression was seen between OLP-E and OLP-R compared to WOSCC (P=0.138 and P=0.206, respectively) (Fig. 1). 

**Fig. 2:**
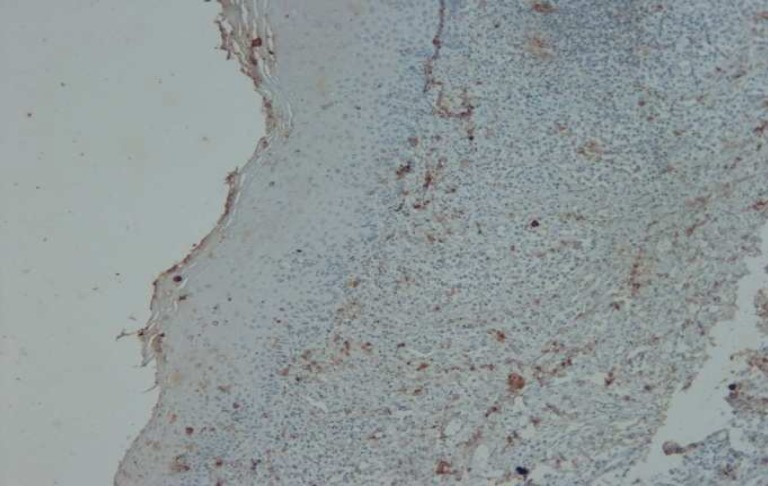
Immunohistochemical staining of Bax in OLP (×100

**Fig. 3:**
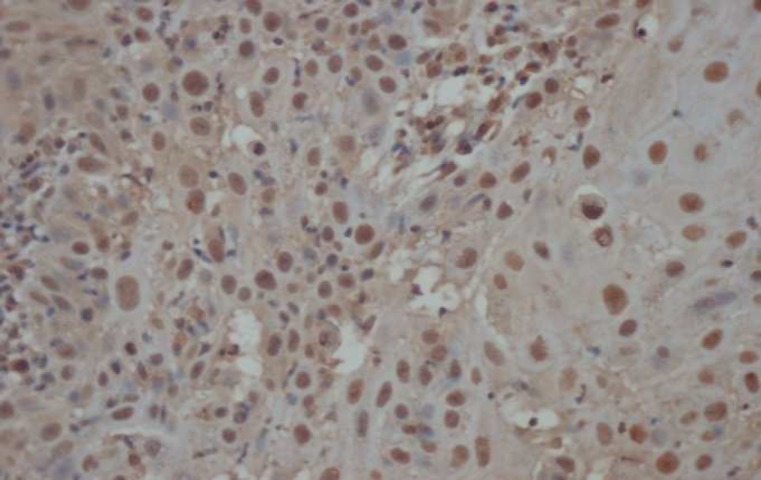
Immunohistochemical staining of Bax in WOSCC (×400

**Fig. 4:**
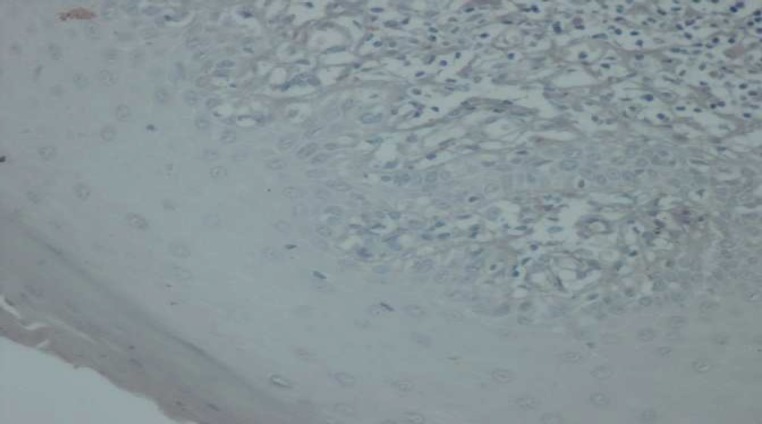
Immunohistochemical staining of Bax in OLP (×100

**Fig. 5:**
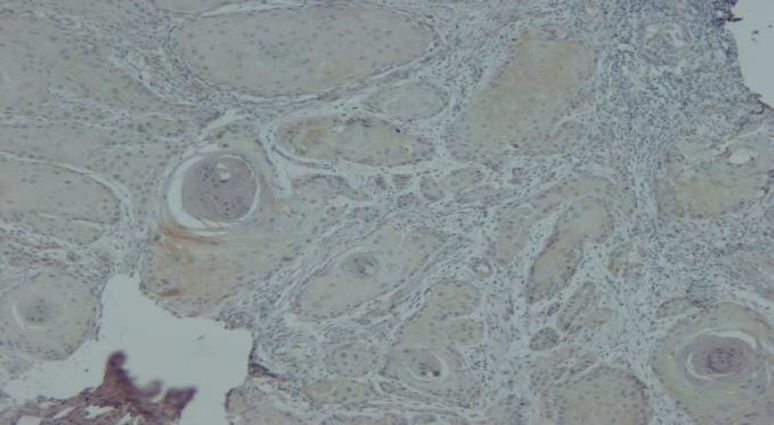
Immunohistochemical staining of Bax in WOSCC (×400

There was no significant difference in bax expression between OLP-E and OLP-R (P=0.91). Bax expression in OLP was significantly higher than normaloral mucosa (P=0.007), but no statistically significant difference between OLP and WOSCC in bax expression level was seen (P=0.089) (Table 1) (Fig. 2, 3). No expression of bcl-2 was seen in OLP and normal mucosa samples. In WOSCC only 4 samples (out of 11) expressed bcl-2 (Fig. 4, 5).

## Discussion

During the last few decades, there has been a large debate on the malignant transformation of OLP and the results are still conflicting. According to WHO definition, a precancerous lesion is a morphologically altered tissue in which cancer is more likely to occur than in its apparently normal counterpart (20-21). The WHO report stated that malignant transformation of OLP is seen in 2-3% of the patients with the disease. This is why the expert group nominated by WHO concluded that OLP is a potentially precancerous condition with a significantly increased risk of cancer. Recently, WHO has stated that the recommended term to be used is “potentially malignant disorder” (22). According to Lee et al., the cell proliferation rate in OLP is higher than in normal mucosa but lower than epithelial dysplasia and squamous cell carcinoma (23). This may explain the reason why WHO states that the malignant transformation rate of OLP is lower than leukoplakia (with epithelial dysplasia) and erythroplakia; as the smaller the cell proliferation rate, the slighter the chance of cells suffering new mutations (6).

**Table 1:** Frequency of Bax positive cells in samples

Groups				Bax
OLP-R	OLP-E	WOSCC	Normal Mucosa	
4	5	0	14	-
7	6	6	6	+
4	4	4	0	++
0	0	1	0	+++

In our study, a significant difference in Bax expression between OLP and normal mucosa was found. This finding was similar to Abdel-Latif et al. (13), Bloor et al. (14) and Bascones-Ilundain et al. studies (24). Chen et al. found bax expression significantly higher than normal mucosa (25), but Sklavounou et al. reported that there was a little difference in Bax expression between normal mucosa and OLP. This difference might be because all their samples were reticular OLP and their control group were epithelial hyperplasia samples (26).

According to our research findings, the expression of bax was significantly different in OLP compared to WOSCC. Bax expression showed no significant differences between OLP-E, OLPR and WOSCC. Sousa et al. found that Bax expression was significantly higher in epithelial dysplasia compared to OLP with a similar Bax expression in OSCC and OLP (6, 27).

This fact can indicate a deregulation of the apoptotic mechanisms in OLP, preventing the death of genetically damaged cells and consequently, increasing the malignant transformation risk (27-28). Some studies suggested that decrease in the expression of bax is essential for the development and progression of oral cancer (16-17, 29-30). 

In 21 cases (70%) of OLP, Bax protein was expressed, but the percentage of cells expressing Bax in OLP-E was not significantly different from OLP-R. Bascones-Ilundain et al. found that the expression of bax in the erosive-atrophic type of OLP was similar to reticular type (24). If atrophic-erosive lesions would be more likely to undergo malignant transformation, as it had been proposed, we could have expected to find differences in Bax expression according to the clinical form of OLP (9, 31-32).

In this study, bcl-2 was not expressed in any of OLP samples, similar to the findings of Sklavounou et al. (26) and Leyva-Huerta et al. (33). Bloor et al. showed that bcl-2 expression was very weak or not present in OLP samples. Although bcl-2 was expressed in OLP and normal mucosa, but there was no significant difference between them (14).

Tissue growth depends on the relative rates of cell proliferation and cell death. Decreasing the percentage of apoptotic cells may contribute to tumor formation and progression by increasing cell survival. Higher bax expression not only compen-sates the increase of the cell proliferation rate but also allows the elimination of cells with irreversible genetic damages, which may reduce the effect of carcinogens on the epithelium (6).

Both antiapoptotic Bcl-2 and proapoptotic Bax belonged to apoptosis regulatory proteins. Bcl-2 can form homodimers (Bcl-2 and Bcl-2) and heterodi-mers (Bcl-2 and Bax). In normal conditions, antiapoptotic Bcl-2 forms heterodimers with proapoptotic Bax and inhibits the activation of Bax. When Bcl-2 is over expressed, Bcl-2 homodimers dominate and cells are protected against death. When Bax is in excess, Bax homodimers are predominant and cells can undergo apoptosis. The ratio of Bcl-2⁄Bax regulates the release of cytochrome C from the mitochondria. The follo-wing activation of the caspase and proteolytic cascade leads to cell death. So, cell apoptosis depends on the ratio of these two proteins (34).

For some authors, the small number of apoptotic cells detected in OLP is explained by the brief duration of the apoptotic process (2-3 h) or by the so-called section plane effect, according to which some of the nuclei of some keratinocytes are not observed due to the histologic sectioning, so that even if they are in apoptosis, apoptosis signs are not detected (13-14, 35). Although these interpretations appear reasonable, we do not consider that they explain the low rate of apoptotic cells in OLP. If a large number of epithelial cells were in apoptosis, a higher rate of apoptotic cells would have been observed in this and other studies, even if the process were brief and some cells did not show their nuclei (13, 24, 36). We consider that the little quantitative importance of apoptosis may be explained by the need to preserve the epithelial structure, which may have major consequences for the capacity for malignant transformation of OLP.

The observation that Bax was more exten-sively distributed than Bcl-2 may be consistent with the concept that Bax regulates apoptosis by forming heterodimers with Bcl-2. In those cases of lichen planus in which Bcl-2 was absent, Bax may have bound with other regulatory proteins, including other members of the Bcl-2–related family, such as Bcl-x, McL-1, Bad or Bak. It is conceivable that in OLP, apoptotic cell death may be controlled by extracellular instead of molecular regulators, such as Fas and its ligand (FasL) (14, 37). Indeed, in a previous study, positive staining for Fas and FasL has been detected in the supra basal layers of OLP, in association with Bcl-x and Bax but in the absence of Bcl-2 (38-39). Thus, the evaluation of other apoptosis molecules seems necessary.

Authors declare no conflict of interest.
